# Supplementary material for: Content-rich biological network constructed by mining PubMed abstracts
Source: BMC Bioinformatics. 2004 Oct 8;5:147. doi: 10.1186/1471-2105-5-147 (PMC528731; doi:10.1186/1471-2105-5-147)
Supplement: Additional File 2 — The original results of the above study (non-essential files are deleted to keep the file size under the limit set by BMC bioinformatics). [file 1471-2105-5-147-S2.bz2 › chilibotAdditionalFile2/dip10/106ID1311321E441/html/TCF3_MYF6.html]

 


 **TCF3** and **MYF6** 
  
Found 8 abstracts in PubMed, retrieved 8.  
 

 What does Google say? 
 PDF only 
| .edu only 

---

**Interactive relationship** (e.g. stimulation, inhibition, etc)

**Neutral relationship**- Here, we report that Pbx Meis1 Prep1 binds DNA cooperatively with heterodimers of E2a  [ **TCF3** ]  and MyoD, myogenin, Mrf 4  [ **MYF6** ]  or Myf 5.  Ref: 10471746 Nucleic Acids Res, 1999
- The muscle specific helix loop helix HLH proteins myogenin, MyoD, myf5, and MRF4  [ **MYF6** ]  form hetero oligomers with ubiquitous HLH proteins encoded by the E2A  [ **TCF3** ]  gene and activate muscle transcription by binding to a DNA sequence known as an E box CANNTG.  Ref: 1317849 J Biol Chem, 1992

**Non-interactive relationship** (e.g. studied together, co-existance, homology, etc.)

- In NIH3T3 fibroblasts, the ubiquitous helix loop helix HLH protein E2A  [ **TCF3** ]  E12 E47 and the myogenic HLH proteins MyoD, MRF4  [ **MYF6** ]  and myogenin are growth inhibitory, while two ubiquitous Id proteins lacking the basic region are not.  Ref: 7925274 EMBO J, 1994
- The myogenic regulatory factors MRFs, MyoD, myogenin, Myf 5, and MRF4  [ **MYF6** ] , function as transcriptional activators of muscle specific gene expression by forming heterodimers with the ubiquitously expressed products of the E2A  [ **TCF3** ]  gene, E12 and E47.  Ref: 9116507 Protein Expr Purif, 1997
